# Endovascular treatment of penetrating thoracic aorta injury – case report

**DOI:** 10.1590/1677-5449.200132

**Published:** 2020-11-16

**Authors:** Lucas Mansano Sarquis, Wilson Michaelis, Antonio Lacerda Santos, Rogerio Akira Yokoyama, Mariana Vieira Delazeri, Antonio Luiz da Costa Martins, Rodrigo Krieger Martins, Bruno Berardi Gazola

**Affiliations:** 1 Hospital do Trabalhador, Curitiba, PR, Brasil.; 2 Hospital Universitário Evangélico Mackenzie, Curitiba, PR, Brasil.; 3 Faculdade Pequeno Príncipe, Curitiba, PR, Brasil.

**Keywords:** thoracic aorta, wounds and injuries, endovascular procedures

## Abstract

In the current scenario, traumas with violent causes are responsible for large numbers of cases. Among these, thoracic aorta injury caused by penetrating trauma is a cause of elevated morbidity and mortality, demanding adequate diagnosis, and can now often be repaired using endovascular procedures. This treatment method has proven to be safer, with a lower rate of complications than open surgical procedures. After endovascular repair, it is necessary to conduct continuous monitoring of the patient’s health and correct any complications related to the procedure that may emerge. The objective of this article is to describe a case of penetrating trauma of the thoracic aorta that was treated endovascularly, since the literature predominantly covers blunt trauma injuries.

## INTRODUCTION

External causes are now the third greatest cause of mortality in Brazil, with predominance of interpersonal violence and traffic accidents, especially in the younger population.^1^ Thoracic traumas can be classified as blunt or penetrating. Penetrating traumas are generally provoked by application of a mechanical force to a small area on the surface of the chest, causing a break in the continuity of the skin and chest wall.^1^ The majority of thoracic aorta ruptures are caused by blunt thoracic trauma,^2^ and, among the most common penetrating injuries, those that involve the aorta are predominantly caused by firearms or cold weapons, in which the quality of pre-hospital care and diagnostic tools is of fundamental importance.^3^ Penetrating injuries involving the thoracic aorta are rare and the majority need to be treated with open surgery, with few applications for endovascular methods, since patients are generally admitted hemodynamically unstable.^4^ Eighty percent of patients with thoracic aorta injuries die at the scene of trauma and seven in every 10 are male.^5^

After stabilization of the patient, diagnosis of aortic injury is a challenge for the surgeon, since in addition to identifying them it is also necessary to treat other associated traumatisms. Concomitant injuries seen in cases of high energy trauma include severe traumatic brain injury (TBI), pulmonary and/or cardiac contusion, abdominal hemorrhage, fractures to long and/or pelvic bones, and ruptured diaphragms.^6^ Symptoms of aortic rupture can include pain in the chest, back, or abdomen, in addition to external signs of thoracic injury.^7^

Diagnostic suspicion begins with physical examination and chest X-ray; but both are of low sensitivity. Computed tomography angiography (angio-CT) is considered the gold standard, since in addition to diagnosis, it can be used to plan endovascular treatment. However, in order to conduct an examination, the patient must be stable from a hemodynamic point of view.^4^ Aortic injuries are classified as grade 1 – intimal lesion; grade 2 – pseudoaneurysm; grade 3 – intramural hematoma; or grade 4 – total rupture.^8^

The first report of thoracic endovascular aortic repair (TEVAR) was published in the 1990s and this has now become the technique that is most used, consisting of deployment of an endograft from a peripheral access. As lower profile devices have been developed with better navigation properties and treating teams have gained experience, complications related to the procedure have reduced.^9^ This method of treatment stands out, especially for traumatic aortic injuries and complicated acute thoracic aorta dissection, because of the lower morbidity and mortality compared with traditional surgical repair.^10^

Given that the majority of studies cover injuries to the aorta caused by blunt traumas, the objective of this article is to highlight the importance of endovascular repair in patients who have suffered penetrating traumas with injuries to the aorta.

## CASE DESCRIPTION

**Figure 1:**
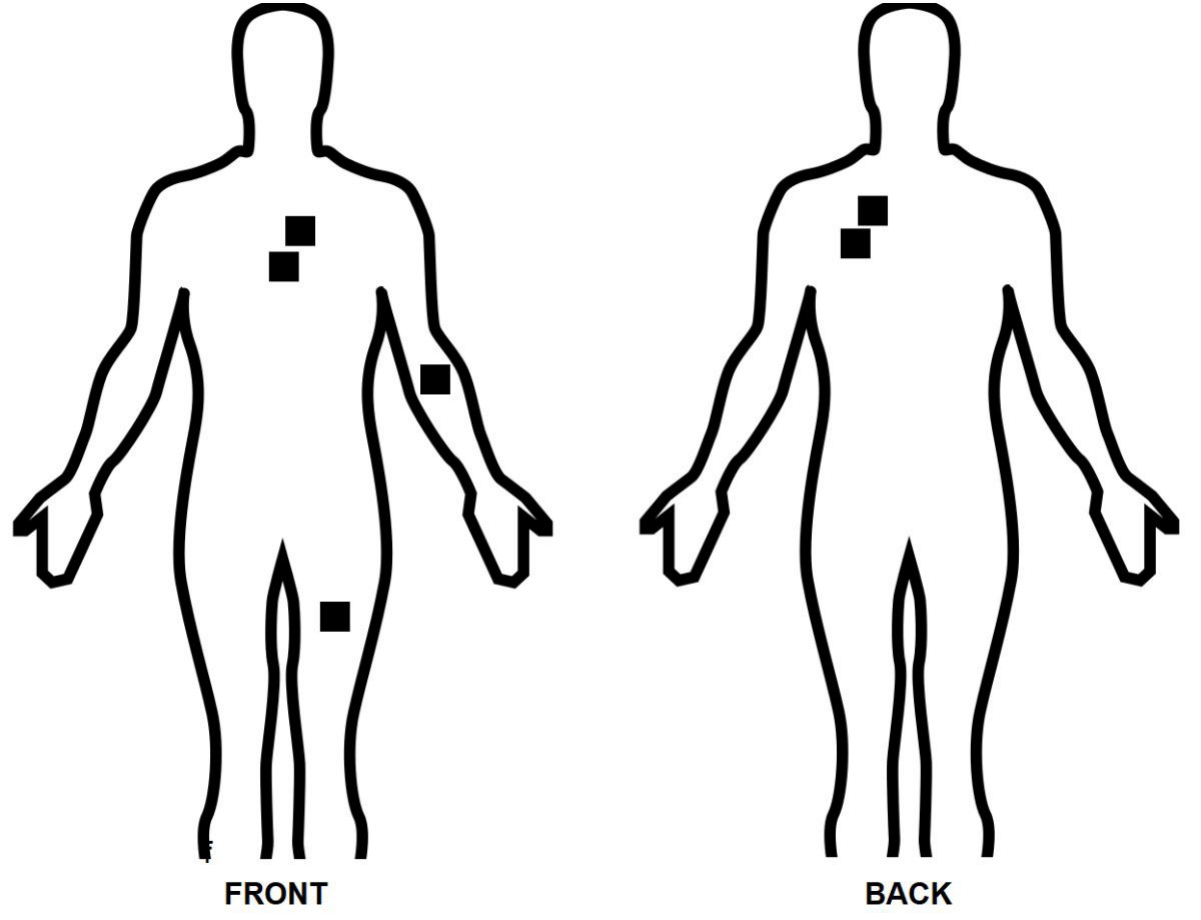
Diagram illustrating cold weapon wound sites.

The patient was a 36-year-old male, admitted to a specialist trauma center on 24 December, 2019, at 21:00, having suffered wounds caused by a cold weapon. He had exhibited hemodynamic instability during transport, but after fluid resuscitation he was stable when he arrived at the hospital. The most notable findings of the physical examination conducted in the emergency room were subdued vesicular murmur in the left hemithorax, heart rate of 125 bpm, mean blood pressure (MBP) of 70-75 mmHg, and multiple wounds to the anterior and posterior chest (Figure 1).

**Figure 2:**
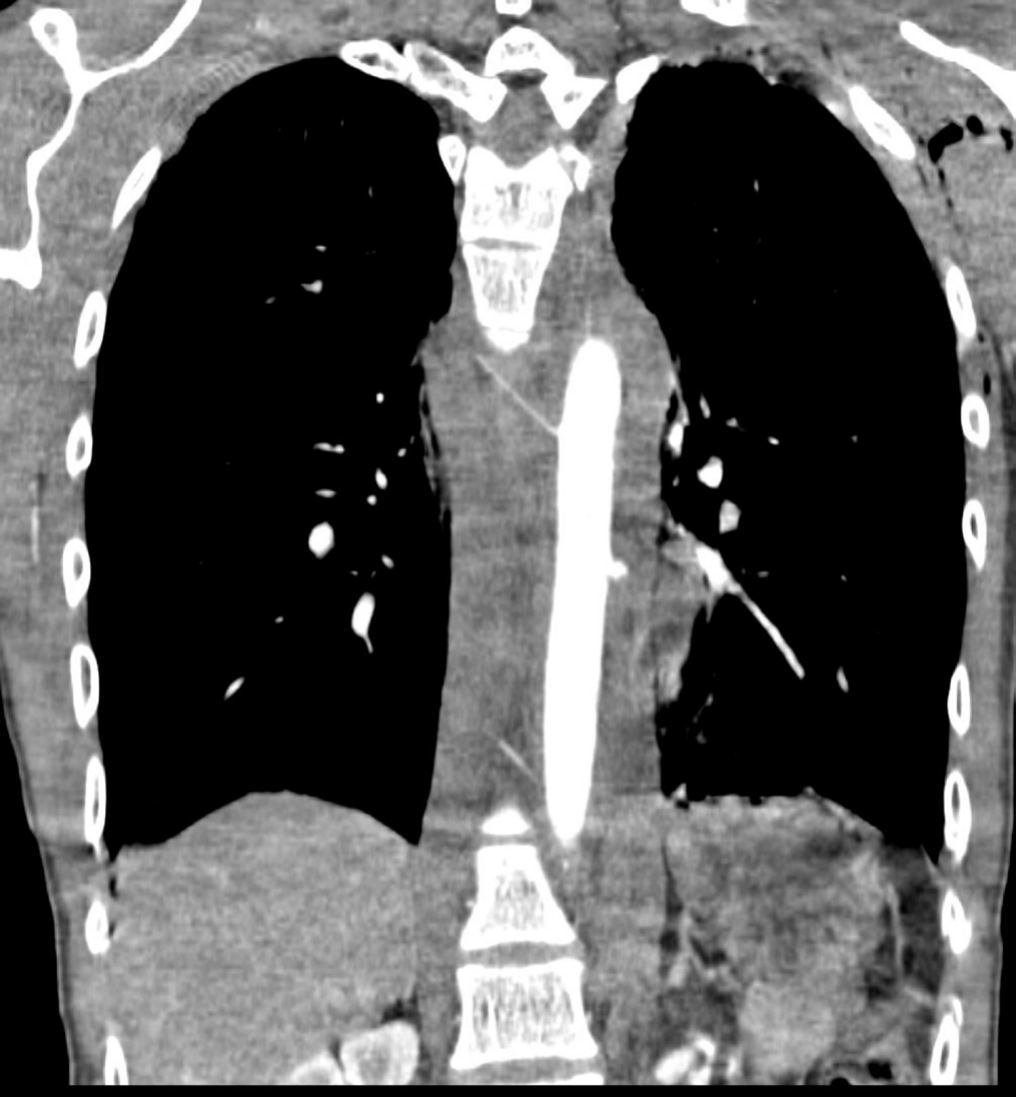
Coronal angio-CT image showing contrast leakage from the thoracic aorta.

**Figure 3:**
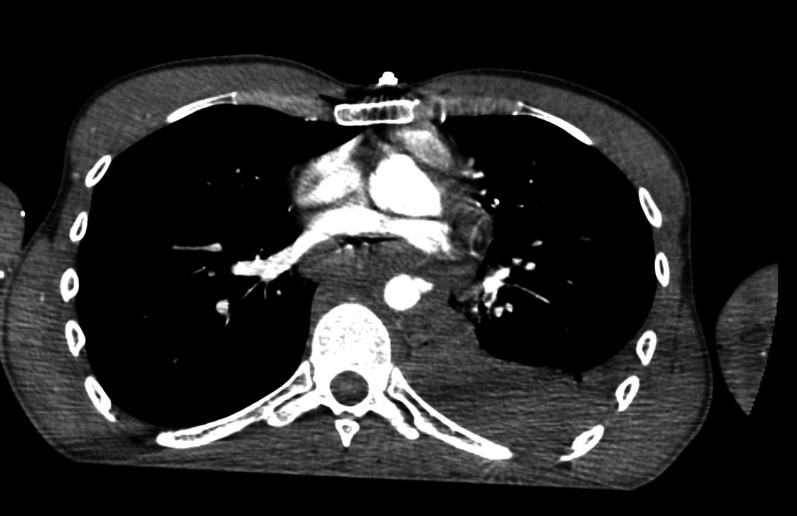
Axial angio-CT image showing contrast leakage from the thoracic aorta.

After administration of packed red blood cells and prophylactic antibiotics, angio-CT of the thorax was conducted (Figures 2 and 3), showing contrast leakage from the thoracic aorta and left sided hemothorax. Left pleural drainage and arteriography of the thoracic aorta were performed.

**Figure 4:**
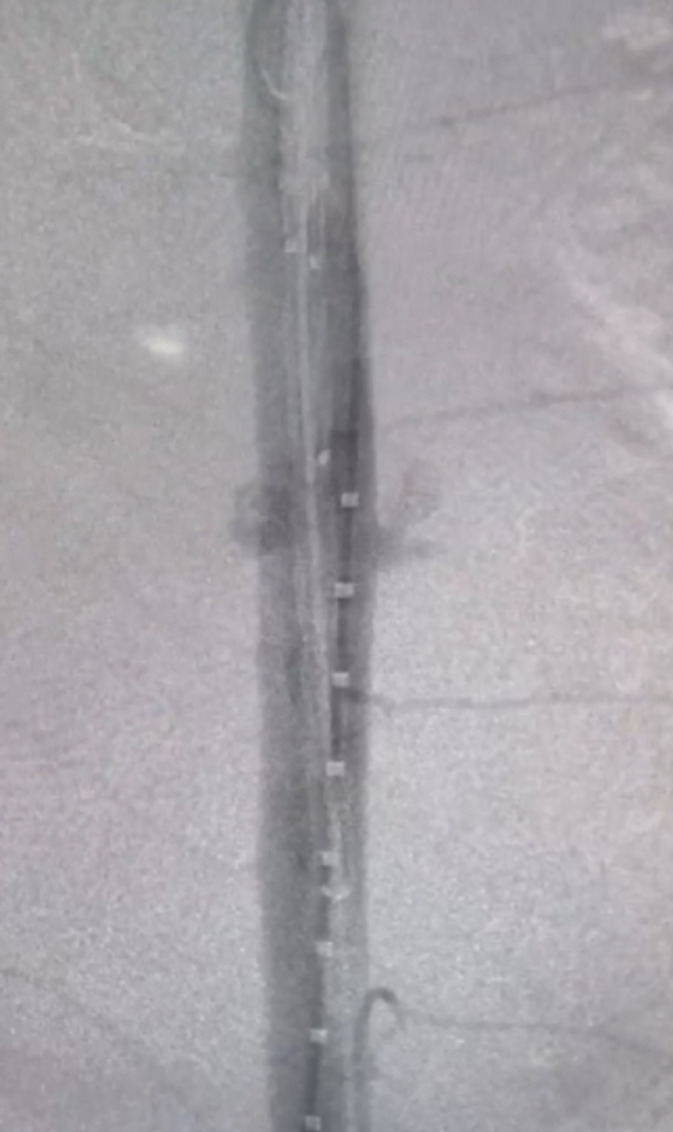
Aortography showing thoracic aorta injury.

**Figure 5:**
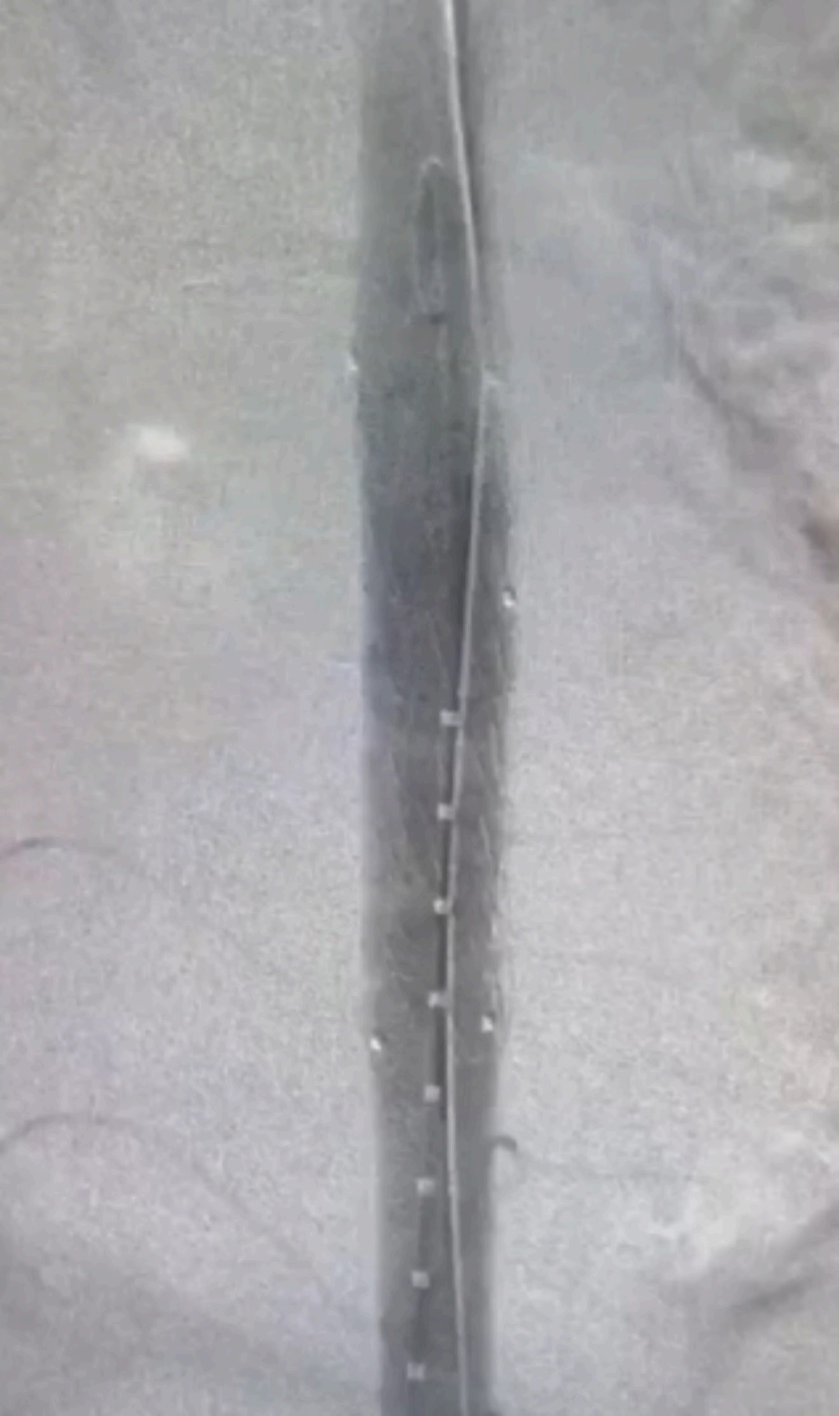
Control aortography after implantation of an endograft.

The decision was taken to proceed directly to definitive endovascular treatment. Intraoperative aortography (Figure 4) was performed, confirming the damage to the thoracic aorta (at the level of T8) with adjacent tamponade, and a 20/20/82 mm straight thoracic endograft was deployed in the descending thoracic aorta. The endovascular intervention was conducted at 01:00 on December 25, 2019. Control aortography performed after implantation (Figure 5) showed no evidence of contrast leakage.

The patient remained in the intensive care unit during the immediate postoperative period. On the first postoperative (PO) day, the patient suffered acute myocardial infarction of the lateral wall, but without compromise to cardiac or hemodynamic function. He was discharged to the wards on PO day 3. The patient had a collection in the left hemithorax due to retained hemothorax, and underwent videothoracoscopy and decortication, conducted by the thoracic surgery team. His recovery was otherwise uneventful and he was discharged from hospital on the 10th day after admission. He is currently in outpatients follow-up with the specialties and is asymptomatic. He had a control chest angio-CT 30 days after the trauma, which found no evidence of complications related to the surgical procedure.

## DISCUSSION

Penetrating injuries to the thoracic aorta are potentially fatal and need medical care that is correctly coordinated, in order to define the order of priority of the care to be provided. Control of blood pressure is one of the first measures, aiming to achieve an MBP in the range of 60-70 mmHg, with systolic pressure below 130 mmHg, which is one of the fundamental factors in ensuring that there is enough time to attempt repair, whether conventional or endovascular.^11^ After initial stabilization of the patient, as in this case, with transfusion of blood products, angio-CT is essential for identification of the aortic injury and assessment of the anatomy to decide on the possibility of endovascular treatment.

Treatment of the aortic injury can be postponed, generally by 1-2 weeks, but sometimes up to 45 days, according to an evaluation made in conjunction with other specialties. In the case of multiple trauma patients, treatment of life-threatening injuries should always be prioritized.^12^ However, early intervention (less than 24 h from hospital admission) should be encouraged for patients with expanding pseudoaneurysms, incapacity to maintain target blood pressure, or leakage of contrast on angio-CT.^12^

Since the patient in the current report did not have other injuries that needed specific treatment, the aortic injury was treated immediately. Concurrent injuries, the grade of aortic injury, hemodynamic stability, and associated comorbidities are the determinant factors of the choice of time and type of repair.^6^ Although the injury was classified as grade 4, total rupture of the aorta combined with hemothorax, the favorable anatomy for endovascular treatment and the patient’s hemodynamic stability made TEVAR a possible option. This aortic repair causes less blood loss, does not require cross-clamping of the aorta, and is associated with faster recovery than open procedures.^13^

The endograft is introduced using guides, via a common femoral artery access, and advanced until it reaches the thoracic aorta, where it is positioned. Essential characteristics of a successful procedure include choosing a prosthesis with appropriate size and shape and appropriate patient vascular anatomy. In healthy patients without prior vascular diseases, which is the situation in the majority of cases, it is possible to reduce the neck of the healthy aorta to 0.5-0.7 cm and seal the endograft, whereas in patients with preexisting vascular disease, the ideal would be to maintain a neck of 1.5-2.5 cm.^11^ The type of device chosen is primarily dependent on the patient’s anatomy, the angles involved, and the stock available for use at the time of trauma.^4^

After implantation, the patient will need lifelong monitoring with imaging exams. Guidelines suggest intervals of 30 days, 6 months, and 12 months after repair and, if there are no complications, annually thereafter.^13^ The open procedure, which requires thoracotomy or sternotomy, is associated with a 28% mortality rate and around 16% paraplegia,^3^ whereas overall 30-day mortality rates linked to TEVAR are around 8%.^14^ Common complications seen after endovascular treatment include endoleaks, graft migration, graft stenosis, and graft infection, in addition to systemic complications such as ischemia, cerebrovascular events, and inflammatory reactions to the material implanted.^13^ Endoleaks are one of the most common indications for reintervention after this type of repair, with incidence of around 12.1%.^10^ Acute myocardial infarction after endovascular treatment for aortic diseases is a rare cause of complications, but can be present.^10^ With regard to the acute myocardial infarction seen in the case described here, we cannot conclude that it was an event that occurred as a result of the endovascular procedure or even because of the penetrating trauma, but not identified during examinations conducted on admission.

It is thus concluded that endovascular treatment of the thoracic aorta is a viable option after penetrating injuries. Prospective and multicenter studies are needed to evaluate long-term results of placement of aortic endografts in patients who have been victims of penetrating wounds.
