# Supplementary material for: Endovascular treatment of penetrating thoracic aorta injury – case report
Source: J Vasc Bras. 2020 Nov 16;19:e20200132. doi: 10.1590/1677-5449.200132 (PMC8217996; doi:10.1590/1677-5449.200132)
Supplement: Translation

# Translation: Abordagem endovascular de lesão de aorta torácica penetrante – relato de caso

**Authors:** Lucas Mansano Sarquis, Wilson Michaelis, Antonio Lacerda Santos, Rogerio Akira Yokoyama, Mariana Vieira Delazeri, Antonio Luiz da Costa Martins, Rodrigo Krieger Martins, Bruno Berardi Gazola

**Affiliations:** 1 Hospital do Trabalhador, Curitiba, PR, Brasil.; 2 Hospital Universitário Evangélico Mackenzie, Curitiba, PR, Brasil.; 3 Faculdade Pequeno Príncipe, Curitiba, PR, Brasil.

**Keywords:** aorta torácica, ferimentos e lesões, procedimentos endovasculares

## Abstract

O cenário atual do trauma por causas violentas tem apresentado um elevado número de casos. Entre eles, a lesão de aorta torácica por trauma penetrante é uma condição de elevada morbimortalidade que exige diagnóstico adequado e que, nos últimos anos, tem sido corrigida com procedimentos endovasculares. Essa modalidade de tratamento se mostra mais segura, com menor número de complicações em relação ao procedimento cirúrgico aberto. Após a realização da abordagem endovascular, há necessidade de acompanhamento contínuo, que visa monitorar as condições de saúde do paciente e corrigir possíveis complicações relacionadas ao procedimento. O objetivo deste artigo é relatar um caso de traumatismo penetrante de aorta torácica, tratado por via endovascular, visto que a literatura aborda predominantemente as lesões por trauma contuso.

## INTRODUÇÃO

Atualmente, as causas externas, em que se destacam a violência interpessoal e os acidentes de trânsito, representam a terceira causa de mortalidade no Brasil, especialmente entre a população jovem^1^. O trauma torácico pode ser classificado em contuso ou penetrante. O penetrante geralmente é provocado por aplicação de força mecânica sobre uma área pequena na superfície torácica, que gera uma solução de continuidade da pele e da parede torácica^1^. A ruptura da aorta torácica é majoritariamente causada por trauma contuso de tórax^2^, e, entre as principais lesões penetrantes, o acometimento da aorta é predominantemente causado por armas de fogo ou por armas brancas, sendo de fundamental importância a qualidade da assistência pré-hospitalar e das ferramentas diagnósticas^3^. As lesões penetrantes de aorta torácica são raras; além disso, na sua maioria, necessitam de abordagem cirúrgica, com pouco espaço para abordagem endovascular, visto que o paciente geralmente é admitido instável hemodinamicamente^4^. Oitenta por cento dos pacientes com lesão da aorta torácica morrem no local do trauma e sete em cada 10 são do sexo masculino^5^.

Após a estabilização do paciente, o diagnóstico das lesões de aorta passa a ser um desafio para o cirurgião, visto que, além de identificá-las, é preciso tratar outros traumatismos associados. Entre as lesões concomitantes nos casos de trauma de alta energia, destacam-se o traumatismo cranioencefálico (TCE) grave, contusão pulmonar e/ou cardíaca, hemorragia abdominal, fraturas de ossos longos e/ou pélvica e ruptura diafragmática^6^. Os sintomas de ruptura aórtica podem se apresentar como dor no peito, no dorso ou no abdome, além de sinais externos de lesão torácica^7^.

A suspeição do diagnóstico se inicia com exame físico e radiografia de tórax; porém, ambos têm baixa sensibilidade. A angiotomografia computadorizada (angio-TC) é considerado o padrão-ouro, visto que, além de fazer o diagnóstico, pode servir para planejar o tratamento endovascular. Entretanto, para a realização do exame é necessário que o paciente esteja estável do ponto de vista hemodinâmico^4^. A classificação das lesões de aorta se divide em: grau 1 - lesão da íntima; grau 2 – pseudoaneurisma; grau 3 – hematoma intramural; e grau 4 – ruptura total^8^.

O primeiro relato de reparo endovascular de aorta torácica (TEVAR) foi registrado nos anos 90, sendo atualmente a técnica mais utilizada, que consiste na colocação de endoprótese a partir de uma localização periférica. Com a evolução do material para perfis mais baixos e com melhor navegação, além da experiência das equipes, houve uma redução de complicações relacionadas ao procedimento^9^. Essa modalidade de tratamento vem se destacando, especialmente para lesões aórticas traumáticas e dissecção aórtica torácica aguda complicada, devido à redução da morbidade e mortalidade em comparação ao reparo operatório tradicional^10^.

Dado que a maioria dos estudos aborda as lesões de aorta por traumas contusos, este trabalho tem o objetivo de ressaltar a importância do reparo endovascular aos pacientes vítimas de trauma torácico penetrante com lesão da aorta.

## DESCRIÇÃO DO CASO

Trata-se de um paciente masculino, 36 anos, admitido em um serviço de referência em trauma no dia 24 de dezembro de 2019 às 21:00 h, vítima de múltiplos ferimentos por arma branca. Ele apresentou instabilidade hemodinâmica durante o transporte, mas, após ressuscitação volêmica, chegou estável ao hospital. Durante o exame físico em sala de emergência, se destacaram murmúrio vesicular reduzido em hemitórax esquerdo, frequência cardíaca (FC) 125 bpm, pressão arterial média (PAM) 70-75 mmHg e múltiplos ferimentos em região anterior e posterior de tórax (Figura 1).

**Figura 1:** Ilustração dos locais com ferimento de arma branca.

Após uso de concentrado de hemácias e antibioticoprofilaxia, optou-se pela realização de angio-TC de tórax (Figuras 2 e 3), a qual identificou extravasamento de contraste em aorta torácica e hemotórax a esquerda. Foi optado por drenagem pleural a esquerda e arteriografia de aorta torácica.

**Figura 2:** Corte coronal de angio-TC identificando extravasamento de contraste em aorta torácica.

**Figura 3:** Corte axial de angio-TC identificando extravasamento de contraste em aorta torácica.

Optou-se por proceder ao tratamento definitivo via endovascular. Durante o procedimento, a aortografia (Figura 4) confirmou a lesão em aorta torácica (nível de T8) com tamponamento adjacente, sendo implantada endoprótese torácica reta 20/20/82 mm em aorta torácica descendente. A abordagem endovascular foi realizada à 01:00 h do dia 25 de dezembro de 2019. O controle pós-implante (Figura 5) não evidenciou extravasamento de contraste.

**Figura 4:** Aortografia com identificação de lesão em aorta torácica.

**Figura 5:** Aortografia de controle após colocação de endoprótese.

O paciente permaneceu em Unidade de Terapia Intensiva no pós-operatório (PO) imediato. No 1° PO, o paciente apresentou infarto agudo do miocárdio (IAM) de parede lateral, porém, sem comprometimento da função cardíaca ou hemodinâmica. No 3° PO, recebeu alta para a enfermaria. O paciente encontrou-se com uma coleção em hemitórax esquerdo por hemotórax retido, sendo submetido a videotoracoscopia e decorticação pela equipe de cirurgia torácica, evoluindo sem demais intercorrências, e recebeu alta hospitalar no 10° dia de internação. O paciente atualmente se encontra em seguimento ambulatorial com as especialidades e assintomático. Já realizou angio-TC de tórax de controle com 30 dias pós-trauma, sem evidências de complicações relacionadas ao procedimento cirúrgico.

## DISCUSSÃO

As lesões penetrantes da aorta torácica são potencialmente fatais e necessitam de assistência médica devidamente coordenada, a fim de elencar a ordem de prioridade dos cuidados a serem prestados. Uma das medidas a serem realizadas no atendimento inicial é o controle da pressão arterial, buscando uma PAM entre 60-70 mmHg e sistólica abaixo de 130 mmHg, sendo um dos fatores fundamentais para permitir que haja tempo hábil até que o reparo, convencional ou endovascular, seja realizado^11^. Após a estabilização inicial do paciente, como neste caso, com a transfusão de hemoderivados, a angio-TC é essencial para identificação da lesão de aorta e avaliação da anatomia para uma possível abordagem endovascular.

O tratamento da lesão de aorta pode ser postergado, geralmente entre 1-2 semanas, podendo chegar até 45 dias, conforme avaliação conjunta com as demais especialidades. Nos casos de pacientes politraumatizados, sempre priorizar o tratamento das lesões que colocam o paciente em risco de vida^12^. Entretanto, a abordagem precoce (em menos de 24 h da admissão hospitalar) deve ser estimulada para pacientes que apresentem aumento do pseudoaneurisma, incapacidade de manter pressão arterial (PA) alvo ou extravasamento de contraste na angio-TC^12^.

Visto que o paciente do presente relato não apresentava demais lesões que demandassem tratamento específico, partiu-se para o tratamento da lesão de aorta. As lesões associadas, o grau da lesão de aorta, a estabilidade hemodinâmica e as comorbidades associadas são fatores determinantes para definir o momento e o tipo de reparo escolhidos^6^. Apesar de a lesão ser classificada como grau 4 – ruptura total da aorta associada com hemotórax, a anatomia favorável para tratamento endovascular e a estabilidade hemodinâmica do paciente possibilitaram a TEVAR. Esse reparo da aorta gera menor perda sanguínea, dispensa o pinçamento cruzado da aorta e tem recuperação mais rápida quando comparado ao procedimento aberto^13^.

A colocação da endoprótese é feita através de guias, com acesso pela artéria femoral comum, até que chegue à aorta torácica, onde é posicionada. Entre as características essenciais para o sucesso do procedimento, está a escolha de uma prótese com tamanho e configuração adequados, bem como a adequação anatômica vascular do próprio paciente. Tratando-se de pacientes saudáveis e sem doenças vasculares prévias, como ocorre na maioria das vezes, é possível diminuir o colo de aorta sadia para selar a endoprótese entre 0,5-0,7 cm, enquanto, para pacientes com doença vascular prévia, o ideal seria manter um colo entre 1,5-2,5 cm^11^. A escolha do tipo de material está relacionada principalmente com a anatomia do paciente, as angulações e a disponibilidade para uso no momento do trauma^4^.

Após o implante, há a necessidade de acompanhamento ao longo da vida por meio de exames de imagem. As diretrizes sugerem periodicidade de 30 dias, 6 e 12 meses após o reparo e, em seguida, anualmente, caso não haja complicações^13^. O procedimento aberto, que necessita de toracotomia ou esternotomia, está associado a uma taxa de 28% de mortalidade e de cerca de 16% de paraplegia^3^, enquanto as taxas de mortalidade geral em 30 dias, relacionadas ao TEVAR, giram em torno de 8%^14^. Entre as complicações comumente encontradas após abordagem endovascular, estão vazamentos (*endoleaks*), migração, estenose e infecção do enxerto, além de complicações sistêmicas como isquemias, eventos cerebrovasculares e reações inflamatórias contra o material implantado^13^. Os *endoleaks* representam uma das indicações mais comuns de reintervenções nesse tipo de reparo, com uma incidência em torno de 12,1%^10^. A presença de IAM pós-tratamento endovascular de doenças aórticas é uma causa rara de complicações, mas que pode estar presente^10^. Sobre o IAM apresentado no caso em questão, não podemos concluir se foi um evento que ocorreu em decorrência do procedimento endovascular ou ainda decorrente do trauma penetrante sem identificação nos exames de admissão.

Conclui-se, portanto, que o tratamento endovascular da aorta torácica para lesões penetrantes é uma alternativa viável. Estudos prospectivos e multicêntricos são necessários para avaliar os resultados em longo prazo da colocação de endoprótese aórtica em casos de pacientes vítimas de ferimentos penetrantes.
